# Spatial Expression and Functional Analysis of Casparian Strip Regulatory Genes in Endodermis Reveals the Conserved Mechanism in Tomato

**DOI:** 10.3389/fpls.2018.00832

**Published:** 2018-06-22

**Authors:** Pengxue Li, Meina Yang, Jiang Chang, Junqing Wu, Fenglin Zhong, Abidur Rahman, Haiyang Qin, Shuang Wu

**Affiliations:** ^1^FAFU-UCR Joint Center and Fujian Provincial Key Laboratory of Haixia Applied Plant Systems Biology, College of Horticulture, Fujian Agriculture and Forestry University, Fuzhou, China; ^2^Department of Plant Bio Sciences, Faculty of Agriculture, Iwate University, Morioka, Japan

**Keywords:** evolution, Casparian strip, tomato, SHORT-ROOT, root, MYB36

## Abstract

Casparian strip (CS) is an impregnation of endodermal cell wall, forming an apoplastic diffusion barrier which forces the symplastic and selective transport of nutrients across endodermis. This extracellular structure can be found in the roots of all higher plants and is thought to provide the protection of vascular tissues. In *Arabidopsis*, a genetic toolbox regulating the formation of Casparian strips has emerged recently. However, Arabidopsis has the stereotypical root which is much simpler than most other plant species. To understand the Casparian strip formation in a more complex root system, we examined CS regulatory pathways in tomato. Our results reveal a spatiotemporally conserved expression pattern of most essential components of CS machinery in tomato. Further functional analyses verify the role of homologous CS genes in the Casparian strip formation in tomato, indicating the functional conservation of CS regulatory cascade in tomato.

## Introduction

Despite varied cell layers in ground tissues, nearly all vascular plants have only one endodermal layer surrounding the transporting vessel tissues in their roots (Lim et al., 2000; Enstone et al., 2003; Cui et al., 2007). Endodermis functions as a barrier between inner stele tissues and outer ground tissues. When endodermal cells of the root begin to mature, a ring-like hydrophobic structure appears on the radial and transverse cell walls, named Casparian strip (CS). This unique structure forms a paracellular transport barrier to disrupt the free diffusion of water and nutrients, enforcing the selective transport between stele and outer cortex via symplastic transport or membrane associated transporters (Heath, 1976; Schreiber and Franke, 2011). The protective role of endodermis is thought to be an adaptation to environmental changes during the terrestrialization (Howard and Bonnett, 1968; Enstone et al., 2003). The emergence of Casparian strip-like structures can be traced back to the root of ferns living about 400 million years ago (Moore et al., 2010; Geldner, 2013).

CS structure was discovered early in 1865 by Robert Caspary (Caspary, 1865), but the molecular basis of CS formation has only emerged in recent years. Naseer et al. (2012) demonstrated that the functional CS is mostly made of the lignin polymer, which overrides the long-standing thought that CS is mostly a suberin-based structure. Moreover, using *Arabidopsis thaliana* as the model system, researchers have identified a large number of essential genes that make the regulatory network of a functional Casparian strip.

In *A. thaliana* root, this lignin strip can be detected in around the 14th endodermal cell above the onset of the elongation zone (Naseer et al., 2012). To ensure the formation of functional CS, two critical regulations are required: (1) spatial mark of CS zone in the middle of the endodermal cells, and (2) specification of the cell layer that is directly contacting stele as the CS forming layer. The localized deposition of lignin relies on a family of transmembrane proteins, the Casparian strip membrane domain proteins (CASPs). CASPs specifically localize at the Casparian strip formation site, guiding the local lignin deposition (Roppolo et al., 2011). It was proposed that CASPs can help to establish the local scaffold to assemble a set of enzymes consisting of Respiratory Burst Oxidase Homolog F (RBOHF), Peroxidase 64 (PER64), Enhanced Suberin 1 (ESB1) and possibly -some other uncharacterized factors (Hosmani et al., 2013; Lee et al., 2013; Kamiya et al., 2015). The precise localization of CASPs was found to be under control of two receptor-like kinases, SCHENGEN (SGN)1 and SGN3 (Alassimone et al., 2016). Both mutants display defects in CS integrity, implying that *SGN1* and *SGN3* play a combinatorial role in the CS formation.

On the tissue level, plants specify the endodermis contacting stele as the cell layer that forms CS by the specific expression of MYB36, a putative master regulator of CS formation (Kamiya et al., 2015; Liberman et al., 2015). In *myb36-1* mutant, CS does not correctly form and a group of CS genes including *CASPs, ESB1*, and *PER64* were downregulated. Interestingly, *MYB36* expression is thought to rely on a GRAS family transcription factor, *SCARECROW* (*SCR)* (Liberman et al., 2015). Based on previous studies, *SCR* itself is the direct target of another GRAS family transcription factor *SHORT-ROOT* (*SHR)* (Helariutta et al., 2000; Paquette and Benfey, 2005; Sozzani et al., 2010). Intriguingly, *SHR* is only expressed in stele from where it moves through cell-to-cell into the neighboring endodermis to play non-cell-autonomous roles, thus providing a positional information during the tissue patterning (Wu and Gallagher, 2014). Recently, another stele-derived signal conveyed by two small peptides, CASPARIAN STRIP INTEGRITY FACTORS1/2 (CIF1/2), was shown to promote the intactness of CS band by interacting with the receptor kinase SGN3 (Doblas et al., 2017; Nakayama et al., 2017), which adds another layer of regulation to the CS formation in *Arabidopsis* roots.

The studies mentioned above have greatly enhanced our understanding of the molecular components involved in CS regulation in *Arabidopsis* roots. However, *Arabidopsis* root has a simple structure, consisting of only three concentric single-celled layers outside of stele (Helariutta et al., 2000; Scheres et al., 2002). In contrast, the roots in most other species have more complex anatomy with multiple cell layers within the ground tissues. Nevertheless, nearly all plants have only one layer of endodermis, indicating a potentially conserved mechanism regulating endodermal cell fate and promoting CS formation in most plant species. A previous study reported that a set of promoters exhibited similar cell type-specificity between *Arabidopsis* and tomato (Ron et al., 2014). In addition, CRISPR-Cas9 based gene knockout in this study demonstrated that the function of *SHR* and *SCR* in root development is presumably conserved in tomato (Ron et al., 2014). This study thus provides the evidence of potentially conserved regulation of root development in tomato. Based on that, we further ask if the components identified in CS formation in *Arabidopsis* function in a conserved manner in other species such as tomato.

In this study, we examined the spatiotemporal expression pattern of most critical regulators in tomato. We showed the spatial expression pattern of crucial CS genes in tomato is very similar to that in *A. thaliana*. Further functional analyses suggest that loss of function of the crucial regulators including *SHR* and *SGN3* results in formation of defective CS. Interestingly, phylogenetic analyses indicate that most critical CS genes maintain high conservation among different plant species, ranging from dicotyledon to gymnosperm and fern. This provides a hint that an evolutionarily conserved mechanism of functional CS formation possibly exists in terrestrial land plants.

## Materials and Methods

### Plant Materials and Growth Conditions

For experiments, *Solanum lycopersicum* cv M82, *Arabidopsis thaliana* cv Columbia and *Glycine max* cv Yue Chun 03-3 were used. The plasmid *pCASP1::CASP1-GFP* was obtained from Dr. Niko Geldner and was transformed into *Arabidopsis* using floral dip method. Seeds were soaked in water for 10 min followed by 75% (v/v) alcohol for 30 s–1 min. Then seeds were sterilized in 50% (v/v) commercial bleach with shaking for 15 min and rinsed with sterile deionized water three times. The seeds were then plated on sterile media (PH = 8.0) containing 0.5 × Murashige and Skoog (MS), 8 g/L agar, 15 g/L sucrose, and 0.5 g/L MES and grown in a 22–25°C incubator with a 16 h light/8 h darkness cycle.

### Phylogenetic Analysis

According to the evolutionary history of the plants, plant species with different evolution stages were chosen, including the dicotyledon plants: *Populus trichocarpa* (Potri), *Medicago truncatula* (Medtr), *Glycine max* (Glyma), *Carica papaya* (Cp), *Arabidopsis thaliana* (AT), *Vitis vinifera* (Vv), *Solanum tuberosum* (St), *Solanum lycopersicum* (Solyc), and *Mimulus guttatus* (Migut), the monocotyledon plants: *Brachypodium distachyon* (Bradi), *Oryza sativa* (LOC_Os), *Zea mays* (Zm), and *Sorghum bicolor* (Sobic), the gymnosperm: *Ginkgo biloba* (Gb) and *Pinus taeda* (Pitae), the ferns: *Selaginella moellendorffii* and (Smo) and the moss: *Physcomitrella patens* (Phpat). The completed amino acid sequences of all plant species were downloaded from Phytozome v12.1^1^. Homologous genes were determined based on PANTHER (Protein ANalysis THrough Evolutionary Relationships) project^2^. Amino acid sequences of selected *Arabidopsis* CS genes as query sequences were obtained from TAIR^3^. We implemented Blastp searches of the complete protein sequences of all species, extracted protein sequences using Blastdbcmd program and performed multiple sequence alignment using Mafft program. The approximately maximum-likelihood phylogenetic trees were constructed by Fast Tree program using Jones-Taylor-Thornton (JTT) model and Shimodaira–Hasegawa test. We beautified the trees on MEGA 7.

### Molecular Cloning

To make GUS reporter lines, we cloned the promoter region (the upstream sequence of the translational start site; 3.3-kb for *SlSHR*, 1.6-kb for *SlMYB36a*, 1.4-kb for *SlPER64a*, 2.2-kb for *SlSGN3b*, 2.3-kb for *SlSGN3a*, and 2-kb for *SlCIF*, respectively) of each gene. The putative promoters were predicted using the promoter prediction software^4^. The tomato promoter fragments were amplified by PCR from M82 genomic DNA (see **Supplementary Table S2** for primers). The DNA fragments were purified through DNA gel extraction kit (TRANS) and were cloned into the HindIII and PsiI site of the linearized expression vector R4L1pGWB432 (AB524006) using In-Fusion system (In-Fusion Exnase^®^ II, Vazyme). *Arabidopsis thaliana* promoters were identified in the same way (see **Supplementary Table S2** for primers) and cloned from *Arabidopsis* (Col.) genomic DNA. The target fragments were then transferred to the expression vector R4L1pGWB632 by In-Fusion Technology (Vazyme Biotech).

For overexpression constructs, tomato genes were amplified from cDNA library of tomato roots by PCR. We modified the expression vector pHellsgate8 by introducing GFP fragment into linearized pHellsgate8 (using EcoRI and XbaI sites) using In-Fusion technique. The gene of interest was cloned into this modified vector by Gateway Cloning Technology (Invitrogen). The primer sequences used for cloning were listed in **Supplementary Table S2**.

For CRISPR constructs, the 19-nucleotide target sites for genes including *SlSHRa, SlMYB36a* and *SlSGN3a* were identified on the website https://www.genome.arizona.edu/crispr/CRISPRsearch.html. The target sequences were blasted against the tomato genome using http://solgenomics.net/tools/blast/ to ensure no other matches were found. Primer sequences used were listed in **Supplementary Table S2**. The target fragments were cloned into linearized (at BsaI site) vector pTX041 (kindly offered by Dr. Chuanyou Li, Key Laboratory of Plant Genomics and National Center for Plant Gene Research, Institute of Genetics and Developmental Biology, Chinese Academy of Sciences, Beijing, China) using In-Fusion technology. The pTX04 vector was derived from pBIN19, in which Cas9 is driven by 2 × 35S and the target sequence was driven by tomato U6 promoter (Ye et al., 2017).

For screening CRISPR-Cas9 knockout lines, positive transgenic lines were verified by PCR for the presence of Cas9. The positive lines were further genotyped for mutations by sequencing the PCR products amplified by a forward primer to the left of sgRNA1 and a reverse primer to the right of sgRNA2. The used primers were: SlSGN3-F/R: GGACCCGTGCTTGACCTTTG/TTTAAGCATAGATAACTCCTCCGGG; SlSHRa-F/R: TTCTAACAAACAAAACAGCACATAC/TCAAAACTAATTTCCTCGTTGACTC; SlMYB36a-F/R: GATTTCAGGGCTTCTGTTTGCTC/GAGAGGCTCAGGCCAACAAGG. Five transgenic lines of each transformation were analyzed. Four lines of *slsgn3a* mutant, three lines of *slshra* mutants, and two lines of *slmyb36a* mutant had the described phenotype.

### *Rhizogenes* Transformation

The hairy-root transformation was conducted according to previous reports (Sun et al., 2006; Ron et al., 2014). Tomato (M82) seeds were soaked, sterilized, and imbibed 6–8 days until cotyledons expanded fully. The expanded cotyledons were cut into 2–3 explants and placed on MS medium (a sterile filter paper was placed on the medium in advance). The explants were placed in dark for 1 day.

For preparation of bacterial suspension, *Agrobacterium rhizogenes* strain K599 was transformed by thermal stimulation method with appropriate plasmids. The transformed strain was cultured in 10 ml of LB medium with antibiotics (50 mg/L streptomycin, 100 mg/L spectinomycin), and incubated at 28°C for 24 h with shaking at 220 rpm until the cells reached mid-log phase. The bacterial suspension was centrifuged at 1, 000 × *g* for 5 min at room temperature and the cell pellets were resuspended in sterile MS liquid medium with 30 g/l sucrose. The explants were infected using the bacterial suspension for 15 min and then grew on MS medium in the dark for 2 days at 25°C followed by growing on antibiotic plates until root emergence. In soybean, we performed hairy-root transformation according to previously reported protocol (Kereszt et al., 2007).

### Staining, Sectioning and Microscopy

GUS staining was performed as described earlier (Lucas et al., 2011; Racolta et al., 2014). The transgenic roots were fixed in 90% acetone for 20 min followed by GUS wash solution containing 100 mM Na_2_HPO_4_/NaH_2_PO_4_ (pH7.0), 1 mM K_3_Fe(CN)_6_, 10 mM Na_2_EDTA, and 0.1% (V/V) Triton-100. The roots were incubated in staining solution (GUS wash solution and 2 mM X-Gluc) at 37°C in dark for 1 h to overnight. For sectioning, the roots were placed into a drop of water on the slide and cut into slices as thin as possible by hand sectioning (Lux et al., 2005). Samples are mounted in HCG (8 chloral hydrate: 3 glycerol: 1 ddH_2_O) for analysis under DIC microscope (Nikon). For CS observation (Peirson and Dumbroff, 1969; Schreiber and Franke, 2011; Zelko et al., 2012), the sections were soaked for 1 h in 0.1% (w/v) solution of berberine (Sigma) in distilled water, washed three times with water, stained for 30 min with an aqueous solution of aniline blue (0.5w/v, Polysciences) and rinsed again. The sections were mounted in 50% glycerol and Casparian strip autofluorescence was detected under UV light (405 nm) with Zeiss LSM880. For *A. thaliana*, the roots were stained by PI (Propidium iodide) and observed under a Zeiss LSM880 confocal microscopy (PI, 561 nm; GFP, 488 nm).

## Results

### Phylogenetic Analysis of Genes Involved in Lignin Deposition

**FIGURE 1:**
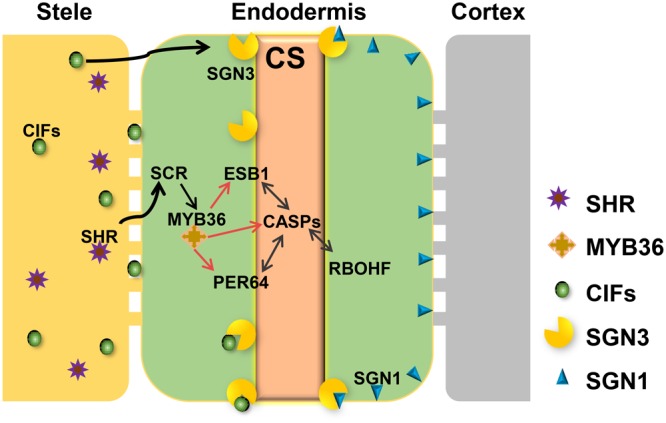
Regulatory network required for Casparian strip formation in Arabidopsis. CASPs act as a local scaffold to recruit *RBOHF, PER64*, and *ESB1* to help local lignin polymerization. MYB36 is a master regulator of activation of the expression of *CASP1, ESB1*, and *PER64*. The expression of *MYB36* depends on *SCR*, a direct target of *SHR*. *SHR* only expresses in stele and moves to the endodermal cell via the symplastic pathway. To precisely restrict CS distribution on the cell membrane, two receptor-like kinases, SGN1 and SGN3, need to play a combinatorial role. The stele derived small peptides, CIFS move to endodermis by apoplastic pathway to bind directly to SGN3 to promote the intact CS. With the coordinative regulation of these components, *A. thaliana* can ensure the precision of spatiotemporal formation of CS in the endodermis directly contacting the stele.

**FIGURE 2:**
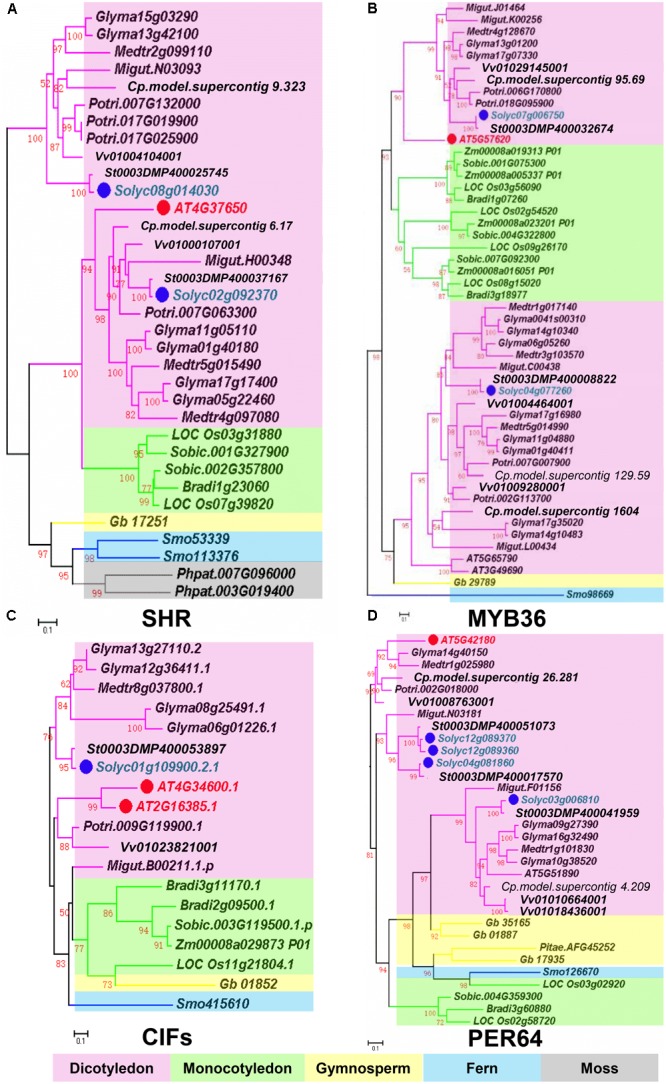
Phylogenetic trees of Casparian strip regulatory genes. **(A)** The phylogenetic tree of *SHR*. **(B)** The phylogenetic tree of *MYB36*. **(C)** The phylogenetic tree of *CIFS*. **(D)** The phylogenetic tree of *PER64*. Red dots show the *Arabidopsis* genes. Green dots highlight homologous genes in *Solanum lycopersicum*. Based on protein sequence alignments, the phylogenetic tree was constructed using FastTree and MEGA7. Only local support values (numbers at the nodes) larger than 50% are indicated. The scale bar is the number of amino acid substitutions. See **Supplementary Table S1** for the species abbreviations.

Based on recent studies on CS regulation in *Arabidopsis*, we illustrate the whole scenario of CS formation in a model (**Figure 1**). To understand the evolution and conservation of CS regulators, we analyzed sequenced plant genomes for these regulators and constructed the phylogenetic trees using the full-length amino acid sequences (**Figure 2** and **Supplementary Figure S1**). In the phylogenetic analysis, we included most essential components of CS regulation identified so far. In the upstream of this regulatory network are a number of transcription factors, including *SHR, SCR*, and *MYB36*. In more downstream are genes including *PER64, SGN1, SGN2, SGN3, SGN4, CASP1*, and *ESB1.* In addition, we also analyzed a recently identified small peptide signaling system (*CIF1* and *CIF2*) that is thought to monitor the intactness of CS. Our phylogenetic trees showed the high conservation of most genes during the evolution among different species ranging from dicotyledon and monocotyledon to gymnosperm and fern. Moreover, *SHR, SCR, SGN1, SGN2*, and *SGN4* were even found in the moss *Physcomitrella patens* (**Supplementary Table S1**).

Based on phylogenetic analysis, we identified the homologous genes of CS regulators in tomato (**Table 1**). The amino acid sequence comparison, revealed a high homology of the CS genes in *Arabidopsis* and tomato. To understand if the highly conserved sequence of CS regulatory genes has any biological significance, we first examined their expression in different tissues using tomato digital expression data^5^ (Tomato Genome Consortium, 2012). Based on the data from the transcriptome analysis of various tissues in tomato cultivar *Heinz* and the wild relative *Solanum pimpinellifolium*, most genes essential for CS formation were found to be highly and specifically expressed in the root (**Supplementary Figure S2**).

**Table 1:** The information of homologous genes in *Solanum lycopersicum* genome.

*S. lycopersicum* gene	Synonym	*A. thaliana* gene	Synonym	Identity(%)	*E*-value
*Solyc02g092370*	SlSHRa	*AT4G37650*	SHR	61.8	0
*Solyc08g014030*	SlSHRb	*AT4G37650*	SHR	51.34	1E-137
*Solyc04g077260*	SlMYB36a	*AT5G57620*	MYB36	91.06	1.00E-77
*Solyc07g006750*	SlMYB36b	*AT5G57620*	MYB36	52.19	2.00E-96
*Solyc12g089360*	SlPER64a	*AT5G42180*	PER64	71.61	8.00E-167
*Solyc12g089370*	SlPER64b	*AT5G42180*	PER64	72.26	2E-167
*Solyc04g081860*	SlPER64c	*AT5G42180*	PER64	67.09	4E-155
*Solyc02g083480*	SlPER64d	*AT5G42180*	PER64	65.15	1E-148
*Solyc01g109900*	SlCIF	*AT2G16385*	CIF1	47.62	6.00E-17
*Solyc01g109900*	SlCIF	*AT4G34600*	CIF2	47.62	6.00E-17
*Solyc08g074980*	SlSGN1a	*AT1G61590*	SGN1	74.01	0
*Solyc05g025820*	SlSGN1b	*AT1G61590*	SGN1	61.47	7e-155
*Solyc11g069520*	SlSGN2	*AT1G08030*	SGN2	56.82	0
*Solyc05g007230*	SlSGN3a	*AT4G20140*	SGN3	63.95	0
*Solyc03g112680*	SlSGN3b	*AT4G20140*	SGN3	47.48	0
*Solyc08g081690*	SlSGN4	*AT1G64060*	RBOHF	77.11	0
*Solyc10g083250*	SlCASP1a	*AT2G36100*	CASP1	55.5	9.00E-68
*Solyc06g074230*	SlCASP1b	*AT2G36100*	CASP1	58.91	3.00E-66
*Solyc09g010200*	SlCASP1c	*AT2G36100*	CASP1	55.14	1E-065
*Solyc04g005620*	SlCASP1d	*AT2G36100*	CASP1	46.37	2E-050
*Solyc06g075630*	SlESB1a	*AT2G28670*	ESB1	64.68	1.00E-94
*Solyc11g073030*	SlESB1b	*AT2G28670*	ESB1	58	4.00E-94
*Solyc10g074680*	SlSCR	AT3G54220	SCR	83.54	0

### The Endodermal Expression of CS-genes in Tomato

**FIGURE 3:**
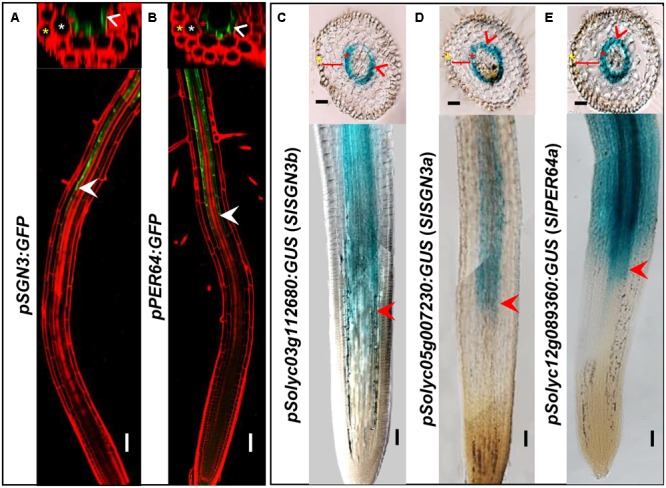
Comparison of gene expression between *Arabidopsis thaliana* and tomato (M82). Tissue and cell type-specific expressions of CS genes (*AtSGN3, AtPER64*) in *Arabidopsis thaliana* **(A,B)**. Red is PI-stained cell wall and green is GFP fluorescence; White arrow heads point to the spatial expressing sites. The asterisks in yellow, white, and red indicate epidermis, cortex, and endodermis, respectively. Endodermis-specific expression conferred by the promoters *SlSGN3b, SlSGN3a*, and *SlPER64a* in the M82 hairy root transformation **(C–E)**. Red arrow heads point to the spatial expressing sites. The cell layers enclosed in braces are cortex. Bars = 50 μm.

In addition to the high level of expression in roots, spatiotemporal expression pattern of the CS regulatory genes can provide the further evidence of their functional conservation. To investigate whether the CS regulation in tomato is consistent with *Arabidopsis thaliana*, we chose a number of critical CS homologous genes in tomato genome and cloned their promoters into the expression vector fused with a *GUS* reporter. *AtPER64* plays an important role in lignin deposition and a receptor kinase AtSGN3 controls the correct localization of CS in the endodermis of *Arabidopsis*. In line with their function, they are specifically expressed in the endodermis (**Figures 3A,B**) (Lee et al., 2013; Pfister et al., 2014). Coincidentally, their homologs in tomato, *Solyc03g112680* (*SlSGN3b*), *Solyc05g007230* (*SlSGN3a*), and *Solyc12g089360* (*SlPER64a*), all have the ability to drive the specific expression of GUS in the root endodermis, starting around the onset of differentiation (**Figures 3C–E**), which is similar to the pattern previously reported in *Arabidopsis* (Lee et al., 2013; Pfister et al., 2014). The spatial expression patterns of CS genes imply the regulation of CS formation may be conserved in tomato.

### Spatial Activation Pattern of Upstream CS Regulators in Tomato

**FIGURE 4:**
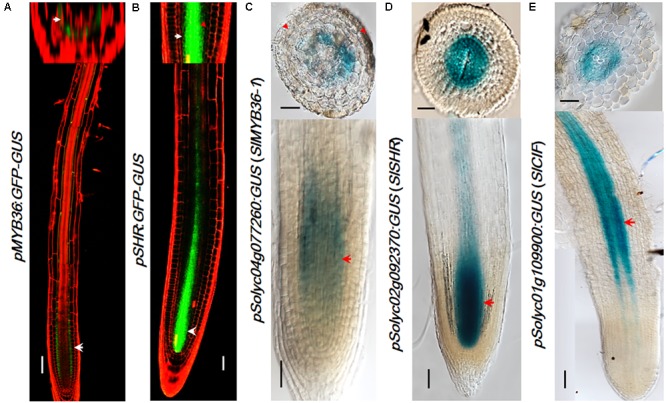
The expression pattern of upstream regulators. *pMYB36::GFP-GUS* expression in *Arabidopsis thaliana* root **(A)**. *pSHR::GFP-GUS* expression in *Arabidopsis thaliana* root **(B).** *SlMYB36apro* fused with GUS protein as the reporter confirms the expression in the endodermis **(C)**. Both the *SlSHRa* promoter **(D)** and *SlCIF* promoter **(E)** drive the expression in vascular tissue in *A. rhizogenes*-transformed hairy roots. Arrowheads point to the expression position. Scale bars = 50 μm.

Many CS catalyzing enzymes are under the direct control of *MYB36*, a transcription factor that is also specifically expressed in the endodermis. Tomato has a MYB36 homologous gene *Solyc04g077260* (*SlMYB36a*) that shows the highest amino acid homology (91.06%) with *Arabidopsis AtMYB36*. To explore *MYB36* expression in tomato, we constructed the reporter lines in which the promoter of *SlMYB36a* was fused with the GUS reporter. Histochemical analysis of GUS expression in tomato roots revealed that *SlMYB36a* promoter is mostly active in the endodermis, which is similar to that of *AtMYB36* (Kamiya et al., 2015) (**Figures 4A,C**).

*SHR* functions at the upstream of *MYB36* of the CS regulatory network. *AtSHR*, specifically expressed in stele (Helariutta et al., 2000) (**Figure 4B**), moves cell-to-cell into the endodermis to play non-cell-autonomous role (Nakajima et al., 2001; Wu et al., 2014). SCR, a direct target of SHR, was reported to regulate the endodermal expression of *AtMYB36* in *Arabidopsis* (Liberman et al., 2015). The expression pattern of SHR and SCR in rice, maize, and tomato were previously found to be similar to that in *Arabidopsis* (Kamiya et al., 2003; Lim et al., 2005; Cui et al., 2007; Ron et al., 2014). Thus it is likely that the function of SHR and SCR in the root radial patterning is well conserved in these species. To verify the SHR expression pattern in the tomato system we examined the promoter activity of *Solyc02g092370 (SlSHRa)*. Similar to the report by Ron et al. (2014), we observed a stele-specific GUS expression in tomato roots transformed with *pSlSHRa:GFP-GUS* (**Figure 4D**).

A parallel signal of SHR is the small peptides, AtCIF1/CIF2, which directly bind to AtSGN3 to promote the intact CS. In *Arabidopsis*, both *AtCIF1/CIF2* are expressed in stele (Doblas et al., 2017; Nakayama et al., 2017). The movement of CIF1/2 from stele to reach SGN3 in the endodermis aids to define the Casparian strip forming layer. Interestingly, we identified only one CIF1/2 homologous gene in tomato (**Figure 2C** and **Supplementary Figure S5A**). *SlCIF* promoter expression was observed in the stele of mature zone of tomato roots (**Figure 4E**). This indicates that the spatial distribution of upstream CS regulators in tomato is also highly conserved.

### Functional Analysis of CS Regulators in Tomato

To further verify the conservation of CS regulators in tomato, we performed functional analysis by gene knock-out and over-expression. One of the most well studied roles of SHR is the promotion of periclinal cell division in *Arabidopsis* (Helariutta et al., 2000; Sena et al., 2004; Wu and Gallagher, 2014). To assess the loss of function effect of SHR in tomato, we used a CRISPR/Cas9 system to knock out *SlSHRa*. A previous study using the same strategy showed loss of function of *SlSHRa* led to short meristematic size (Ron et al., 2014). Here we further examined the root radial patterning which was often used as a marker for SHR functionality. The SHR editing was confirmed by PCR followed by sequencing (**Supplementary Figure S4**).

**FIGURE 5:**
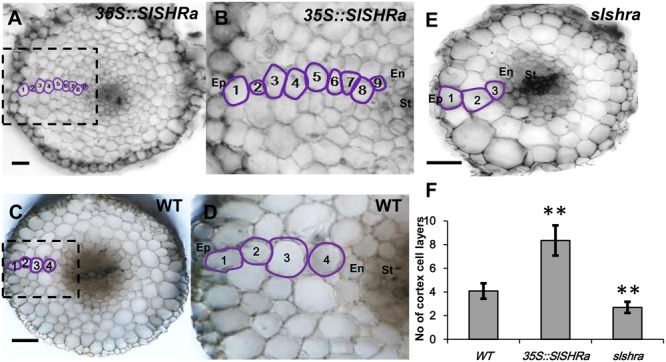
Cortex cell layer number in the root of *35S::SlSHRa* **(A,B)**, WT (transformed with empty vectors) **(C,D)** and the mutant *slshra* **(E)**. **(B,D)** show the zoomed views of boxed regions in **(A)** and **(C)**, respectively. Ep, epidermis; En, endodermis; St, stele; 1 to 9, Cortex cell layers 1 to 9; Bars = 50 μm. **(F)** Quantification of cortex cell layers in **(A,C,E)**. *n* ≥ 20 roots. Error bars are SD of different root cell layers. Significance was determined by Student’s *t*-test, ^∗∗^P < 0.01 (for source data see Supplementary Table S3).

In the cross-sections of *slshra* root, ground tissue layers decreased compared to those in wild-type (WT) (**Figures 5E,F**), indicating the involvement of *SlSHRa* in root radial patterning. Consistent with this, overexpression of SHR in tomato roots resulted in supernumerary ground tissue layers (**Figures 5A–D**). These results indicate that *SlSHRa* is not only essential but sufficient for periclinal cell division in tomato roots.

**FIGURE 6:**
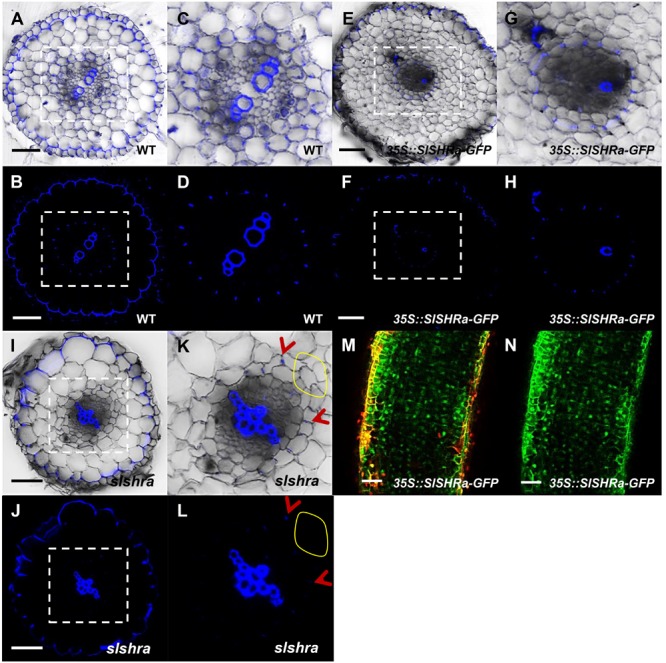
CS staining in WT **(A–D)**, *35S::SlSHRa* **(E–H)**, and *slshra* **(I–L)**. The sections were stained in an aqueous solution of berberine, and post-stained with toluidine blue. The images were taken on the fluorescence microscope under UV light. **(C,D,G,H,K,L)** show the zoomed views of boxed regions in **(A,B,E,F,I,J)**, respectively. Blue dots indicate positions of CS. Red arrow heads point to CS. The areas defined by yellow circles indicate the region lacking CS. Sections in WT, *35S::SlSHRa*, and *slshra* are in similar positions. **(M,N)** show the expression of *35Spro*-driven *SHR* with GFP fusion protein (green) in the transformed root. Bars = 50 μm.

To know if the change of ground tissue layers affects CS formation pattern, we performed lignin auto-fluorescence staining of CS. Compared to continuous CS bands in almost all endodermal cells in WT, *slshra* roots displayed only occasional lignin deposition at the CS position of endodermis (**Figures 6A–D,I–L**). However, in the tomato root with *SlSHRa* overexpression, only one cell layer that is in direct contact with stele formed lignified CS band (**Figures 6E–H**). These observations support the previous report that SHR alone is not sufficient to define the endodermal identity (Wu et al., 2014). The expression of *SlSHRa* was confirmed by the nuclear localized GFP signals (**Figures 6M,N**).

**FIGURE 7:**
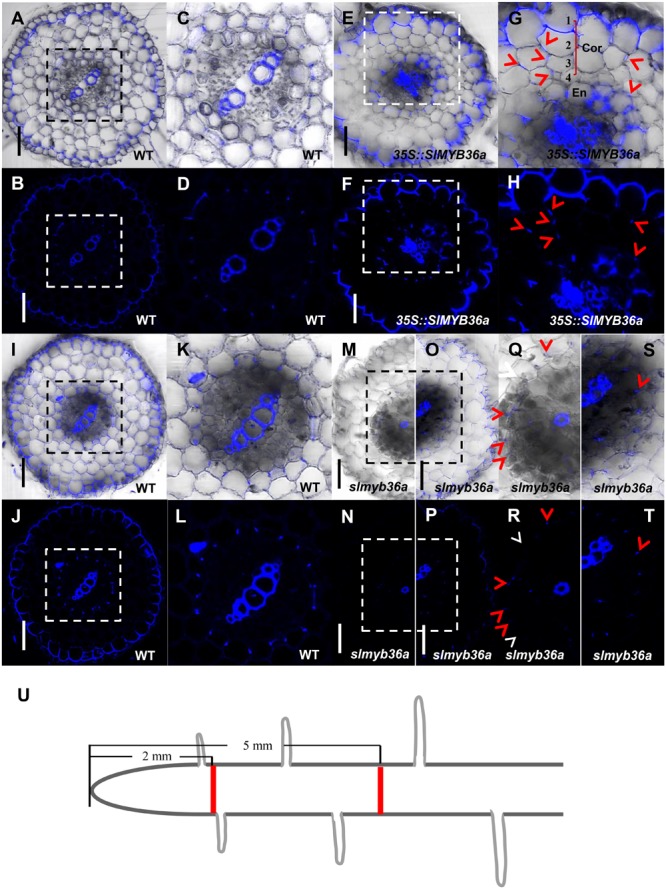
T The function of *SlMYB36a* in CS. **(A–L)** Lignin staining of CS in WT and 35S::*SlMYB36a* roots. En, endodermis; Cor, cortex. Blue dots indicate positions of CS. **(M–T)** show the lignin staining of CS at different positions of *slmyb36a* root. Red arrow heads point to CS; white arrow heads point to the defect of CS. **(I–L,O,P,S,T)** show Casparian strip at 5 mm from the root tip. **(A–D,M,N,Q,R)** show Casparian strip at 2 mm from the root tip. **(Q-T)**, respectively show the zoomed views of boxed regions in **(M-P)**. Schematic showing the sampling position of selection **(U)**. Bars = 50 μm.

In addition to *SHR*, we constructed the over-expression and gene knockout lines for *SlMYB36a*. In over-expressed *SlMYB36a* line, most of the cortical cells had no lignin in. However, presence of discontinuous lignin deposition was found occasionally in a number of cortical cells (**Figures 7E–H**). This observation is consistent with the lignin phenotype found in ectopically expressed *AtMYB36* in *Arabidopsis* (Kamiya et al., 2015). In *Arabidopsis myb36* mutant, CS formation was not entirely abolished but delayed (Kamiya et al., 2015; Liberman et al., 2015). In agreement with this observation, we also found that lignin staining in CS was discontinuous and mostly invisible in the cross-section around 2 mm from the root tip of *slmyb36a* (**Figures 7M,N,Q,R**), although the same position of the WT root exhibited continuous lignin staining of CS (**Figures 7A–D**). However, the *slmyb36a* mutant eventually formed lignin-based CS structure in endodermis around 5 mm from the root tip, indicating the delayed formation of CS (**Figures 7I–L,O,P,S,T**).

**FIGURE 8:**
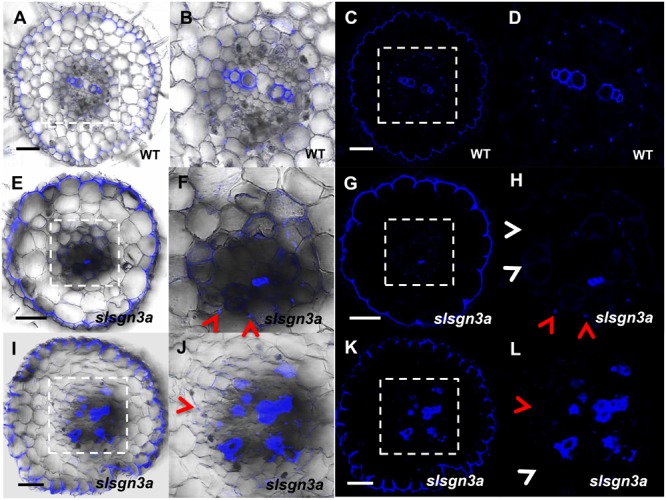
The function of *SlSGN3a* in CS. CS observation in WT **(A–D)** and the mutant *slsgn3a* **(E–L)**. **(B,D,F,H,J,L)** show the zoomed views of boxed regions in **(A,C,E,G,I,K)**, respectively. Blue dots indicate positions of CS. Red arrow heads point to CS; White arrow heads point to the defect of CS. Sections in WT and *slsgn3a* are in similar positions. Bars = 50 μm.

To further understand the pathway of CS formation in tomato, we knocked out *SlSGN3a* in tomato roots and observed discontinuous patches of CS, compared to WT (**Figures 8A–L**). This phenotype mimics the defective CS observed in the *Atsgn3* mutant in *Arabidopsis* (Pfister et al., 2014), suggesting *SGN3* also plays a conserved role in the CS development. Taken together, these results support the idea that the regulatory components for CS formation are functionally conserved among plant species.

### Promoter Activation Is Conserved Among Species

**FIGURE 9:**
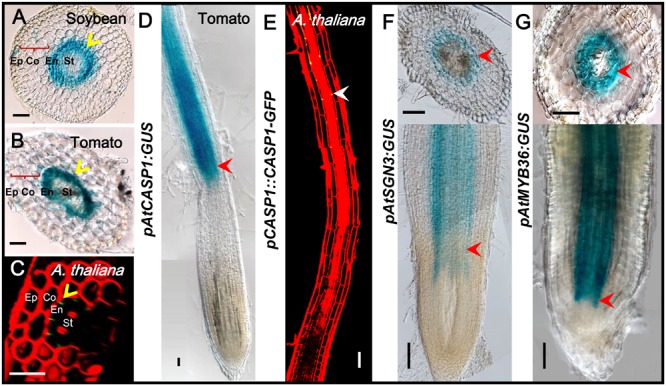
The same expression pattern-conferred by *Arabidopsis* promoters in tomato and soybean. GUS staining shows that *ATCASP1pro* drives the similar expression in endodermal cells of soybean and tomato hairy roots **(A,B)**. *PCASP1::CASP1-GFP* protein fusion shows the localization of CASP1 protein in *Arabidopsis* endodermis **(C,E)**. Ep, epidermis; Co, cortex; En, endodermis; St, stele. The cell layers enclosed in braces are cortex. Yellow arrows point to where genes are expressed. Tomato hairy roots transformed with *ATCASP1pro:GUS* **(D)**, *ATSGN3pro:GUS* **(F)**, and *ATMYB36pro:GUS* **(G)** show specific expression in endodermis. Red arrowheads point to the expression position. Image stitching was performed to show the full expression pattern in the longitudinal view. Bars = 50 μm.

The previous study has confirmed that the expression and localization of CASP1 remains conserved during evolution (Roppolo et al., 2014). The spatiotemporal expression information is mostly included in the *cis*-element of the promoter of a gene. To understand if there are conserved elements in the promoter of CS genes, we cloned the promoter of *Arabidopsis* CS genes and examined their expression pattern in other species. We first constructed *AtCASP1* promoter GUS reporter line and transformed both tomato (M82) and soybean. The promoter fragment (1160 bp of 5′-upstream region preceding the start codon) we used drove the expression specifically in the endodermis of *Arabidopsis* root (**Figures 9C,E**) (Roppolo et al., 2011). Similar to this pattern, we found *AtCASP1* promoter was activated only in the endodermis and mature zone in Tomato and soybean (**Figures 9A,B,D**). We also tested other promoters of CS regulating genes (*AtSGN3, AtMYB36*) that drive endodermis-specific expression in *Arabidopsis* (**Figures 3A, 4A**), and found a similar expression pattern in tomato (**Figures 9F,G**). These results suggest that the transcriptional activation elements within these promoters are possibly conserved across species. Interestingly, by comparing the promoter sequence for each CS gene from different species, we found some common cis-acting regulatory elements whose relative positions are conserved in different promoter regions (**Supplementary Figures S5B–D**).

## Discussion

Formed together with vascular tissues during evolution (Heath, 1976; Enstone et al., 2003; Yoon et al., 2015), Casparian strips are present in ferns, gymnosperms and angiosperms, and some pinales (Warmbrodt and Evert, 1979; Pant and Basu, 1997; Wu et al., 2005). The hydrophobic feature of Casparian strips makes it a protective structure for vascular tissues in plants which help to defend against various environmental stresses (Stasovski and Peterson, 1993; Karahara et al., 2004; Chen et al., 2011; Pfister et al., 2014; Yang et al., 2015; Yoon et al., 2015; Kamiya et al., 2015; Liberman et al., 2015; Liska et al., 2016; Le Roy et al., 2017; Li et al., 2017). However, understanding of molecular regulation behind Casparian strip development is still limited in *Arabidopsis*, in which stereotypical root anatomy is simpler than most other species. Recently, Roppolo et al has shown the conserved subcellular localization of CASP family proteins across species (Roppolo et al., 2014). Upstream regulation of CASPs are mediated by transcription factors while the downstream is regulated by a group of enzymes and factors catalyzing the local lignification. In addition, correct localization of CASPs requires the coordination of two receptor kinases, and intact band-structure of CASPs needs stele-derived peptides, CIF1 and CIF2 (Doblas et al., 2017; Nakayama et al., 2017). In *Arabidopsis* root, both *SHR* and *CIF1/2* are expressed in stele, but the protein and peptides move outward to endodermis to promote Casparian strip development. This intercellular signaling ensures the formation of Casparian strip only in the first cell layer contacting stele. In endodermal cells, *SCR*, the direct target of SHR, can activate the expression of a Casparian strip master regulator, *MYB36*, which turns on the specific expression of a group of downstream regulators, including *CASPs, ESBs and PERs* (Kamiya et al., 2015; Liberman et al., 2015) In addition, SGN1 and SGN3, two kinases that determine the localization of CASPs to CS zone also are specifically expressed in the endodermis. Therefore, spatial activation of these genes is crucial for building the CS only in the endodermis. Phylogenetic analysis revealed the presence of the homologs of these CS regulatory genes in most land plants. However, their functional roles and spatial expression patterns remain obscure in other species. Molecular and cellular analyses in the current study revealed that *SlMYB36a, SlSGN3a* as well as *SlPER64a* have specific expression in the innermost ground tissue layer, while *SlSHRa* and *SlCIF* only expressed in vascular tissues. This finding provides a strong evidence for existence of similar regulatory cascade across different tissues in tomato.

CS is an exclusive feature of endodermal cells and SHR/SCR pathway was proposed to represent an evolutionarily conserved mechanism defining a single endodermal layer in plants (Cui et al., 2007). The rice and maize *SCR* ortholog was also shown to function similarly as *AtSCR* in *Arabidopsis* (Lim et al., 2000, 2005; Kamiya et al., 2003). In tomato, it was shown that loss function of SHR or SCR resulted in short root length and reduced meristem, consistent with their roles in root development in *Arabidopsis* (Ron et al., 2014). Previously it was proposed that SHR is sufficient to confer endodermal identity in root patterning. However, recent studies suggest that SHR alone is not able to reprogram a non-endodermal lineage into a functional endodermis (Wu et al., 2014). In *Arabidopsis*, SHR directly activates *SCR*, and SCR was shown to promote *MYB36* expression, by which a group of Casparian strip genes can be activated. Moving from stele into endodermis to play non-cell-autonomous roles, SHR provides the positional information to specify the cell layer that forms the Casparian strip (Martinka et al., 2012). In tomato, we overexpressed *SlSHRa* and found the increased ground tissue layers. However, we still detected a single lignified cell layer locating in the endodermal position. This verifies that SHR mediated pathway is not adequate to build a functional CS and additional SHR-independent pathways need to participate in the regulation. In contrast, loss-of-function of *SlSHRa* impaired the CS formation in all endodermal cells in mature zone. Interestingly, *slshra* mutant did not entirely lose CS and endodermal cell layer, which is different from the *shr* mutant in *Arabidopsis*. In tomato, we found another *SHRb* gene. It is possible that there is a degree of functional redundancy of these genes in tomato.

The defect in CS formation in *slsgn3a* and *slmyb36a* shows a similar pattern observed in the loss of function Arabidopsis mutants. Additionally, transcriptional activating elements in the promoter and the cis-acting elements in the promoter of many CS regulatory genes were found to be evolutionary conserved among plant species, including tomato. The better way to detect the spatiotemporal change of Casparian strips is the longitudinal observation. However, our attempts of longitudinal examination of Casparian strips in tomato did not work. Due to difference in the root thickness, the methods used in *Arabidopsis thaliana* may not be feasible to use for other species. A number of previous studies using clearing methods also showed that only epidermis and exodermis were clearly visible in the longitudinal section. In those studies, Casparian strips could only be detected in cross sections (Lucas et al., 2011; Lux et al., 2005).

Taken together, our results provide strong functional evidence of the presence of a conserved regulatory mechanism of CS formation in tomato and also suggest that this process is possibly conserved among the land plant species.

## Author Contributions

SW and PL designed the research. PL, MY, JW, and HQ performed the experiments. PL, JC, FZ, and SW analyzed the data. PL, AR, and SW wrote the paper.

## Conflict of Interest Statement

The authors declare that the research was conducted in the absence of any commercial or financial relationships that could be construed as a potential conflict of interest.

## Footnotes

1 https://phytozome.jgi.doe.gov/pz/portal.html

2 http://www.pantherdb.org/

3 http://www.Arabidopsis.org/

4 http://www.cbs.dtu.dk/services/Promoter/

5 http://ted.bti.cornell.edu/cgi-bin/TFGD/digital/home.cgi

## Supplementary Material

FIGURE S1 Phylogenetic trees of Casparian strip regulatory genes *SGN1* **(A)**, *SGN2* **(B)**, *SGN3* **(C)**, *SGN4* **(D)**, *CASP1* **(E)**, *ESB1* **(F)** and *SCR* **(G)**. See Supplementary Table S1 for the species abbreviations.

FIGURE S2 Normalized expression of genes in different tissues of tomato.

FIGURE S3 The *MYB36a* transcript levels in WT and *35S::SlMYB36a* roots using qRT-PCR. Asterisks indicate significant differences from WT, ^∗∗^ P < 0.01, Student’s t test. Values represent the means and error bars indicate SD of the mean from three replicates.

FIGURE S4 Alignment of sequences with Cas9-induced mutations obtained from roots transformed with CRISPR/Cas system for *SlSGN3a* **(A)**, *SlMYB36a* **(B)** and *SlSHRa* **(C)**. Designed target sequences are labeled in the red boxes. The sequences of mutant lines (line 1, line 2) are shown underneath the wild type (WT). The single guide RNA (sgRNA) is marked with red line and mutations are indicated by the blue arrowheads. The changes in length compared with the wild type (*SlSGN3a*: 3753 bp; *SlMYB36a*: 978bp; *SlSHRa*: 1524bp) are shown in red circles on the right.

FIGURE S5 Amino acid sequence alignment of the peptides between *Arabidopsis thaliana* and tomato **(A)**. The homology sequences are shaded in watermelon red. Conservation of promoter regions of *CASP1* homologs **(B)**, *SGN3* homologs **(C)** and *MYB36* homologs **(D)**. *Cis*-acting regulatory sites present in a promoter sequence were searched using PlantCARE.

FIGURE S6 Cell type-specific expression in tomato conferred by the *Solanum lycopersicum* and *Arabidopsis* promoters.

TABLE S1 Numbers of homologous genes in different plant species. Species abbreviations are as follows: Potri, *Populus trichocarpa*; Medtr, *Medicago truncatula*; Glyma, *Glycine max*; Cp, *Carica papaya*; AT, *Arabidopsis thaliana*; Vv, *Vitis vinifera*; St, *Solanum tuberosum*; Solyc, *Solanum lycopersicum*; Migut, *Mimulus guttatus*; Bradi, *Brachypodium distachyon*; LOC_Os, *Oryza sativa*; Zm, *Zea mays*; Sobic, *Sorghum bicolor*; Gb, *Ginkgo biloba*; Pitae, *Pinus taeda*; Smo, *Selaginella moellendorffii*; Phpat, *Physcomitrella patens*.

TABLE S2 Primers used for molecular cloning.

TABLE S3 Cell layers in WT, *35S::SlSHRa*, and *slshra*.

## References

[B1] Alassimone J. Fujita S. Doblas V. G. Van Dop M. Barberon M. Kalmbach L. (2016). Polarly localized kinase SGN1 is required for Casparian strip integrity and positioning. *Nat. Plants* 2:16113. 10.1038/nplants.2016.113 27455051

[B2] Caspary R. (1865). Bemerkungen über die schutzscheide und die bildung des stammes und der wurzel. *Jahrb. Wiss. Bot.* 4 101–124.

[B3] Chen T. Cai X. Wu X. Karahara I. Schreiber L. Lin J. (2011). Casparian strip development and its potential function in salt tolerance. *Plant Signal. Behav.* 6 1499–1502. 10.4161/psb.6.10.17054 21904117 PMC3256377

[B4] Cui H. Levesque M. P. Vernoux T. Jung J. W. Paquette A. J. Gallagher K. L. (2007). An evolutionarily conserved mechanism delimiting SHR movement defines a single layer of endodermis in plants. *Science* 316 421–425. 10.1126/science.1139531 17446396

[B5] Doblas V. G. Smakowska-Luzan E. Fujita S. Alassimone J. Barberon M. Madalinski M. (2017). Root diffusion barrier control by a vasculature-derived peptide binding to the SGN3 receptor. *Science* 355 280–283. 10.1126/science.aaj1562 28104888

[B6] Enstone D. E. Peterson C. A. Fengshan M. (2003). Root endodermis and exodermis: structure, function, and responses to the environment. *J. Plant Growth Regul.* 21 335–351. 10.1007/s00344-003-0002-2

[B7] Geldner N. (2013). The endodermis. *Annu. Rev. Plant Biol.* 64 531–558. 10.1146/annurev-arplant-050312-120050 23451777

[B8] Heath M. C. (1976). Ultrastructural and functional similarity of the haustorial neckband of rust fungi and the Casparian strip of vascular plants. *Can. J. Bot.* 54 2484–2489. 10.1139/b76-266

[B9] Helariutta Y. Fukaki H. Wysocka-Diller J. Nakajima K. Jung J. Sena G. (2000). The SHORT-ROOT gene controls radial patterning of the *Arabidopsis* root through radial signaling. *Cell* 101 555–567. 10.1016/S0092-8674(00)80865-X 10850497

[B10] Hosmani P. S. Kamiya T. Danku J. Naseer S. Geldner N. Guerinot M. L. (2013). Dirigent domain-containing protein is part of the machinery required for formation of the lignin-based Casparian strip in the root. *Proc. Natl. Acad. Sci. U.S.A.* 110 14498–14503. 10.1073/pnas.1308412110 23940370 PMC3761638

[B11] Howard T. Bonnett J. (1968). The root endodermis: fine structure and function. *J. Cell Biol.* 37 199–205. 10.1083/jcb.37.1.199 5645843 PMC2107388

[B12] Kamiya N. Itoh J. Morikami A. Nagato Y. Matsuoka M. (2003). The SCARECROW gene’s role in asymmetric cell divisions in rice plants. *Plant J.* 36 45–54. 10.1046/j.1365-313X.2003.01856.x 12974810

[B13] Kamiya T. Borghi M. Wang P. Danku J. M. Kalmbach L. Hosmani P. S. (2015). The MYB36 transcription factor orchestrates Casparian strip formation. *Proc. Natl. Acad. Sci. U.S.A.* 112 10533–10538. 10.1073/pnas.1507691112 26124109 PMC4547244

[B14] Karahara I. Ikeda A. Kondo T. Uetake Y. (2004). Development of the Casparian strip in primary roots of maize under salt stress. *Planta* 219 41–47. 10.1007/s00425-004-1208-7 14986139

[B15] Kereszt A. Li D. Indrasumunar A. Nguyen C. D. Nontachaiyapoom S. Kinkema M. (2007). Agrobacterium rhizogenes-mediated transformation of soybean to study root biology. *Nat. Protoc.* 2 948–952. 10.1038/nprot.2007.141 17446894

[B16] Le Roy J. Blervacq A. S. Creach A. Huss B. Hawkins S. Neutelings G. (2017). Spatial regulation of monolignol biosynthesis and laccase genes control developmental and stress-related lignin in flax. *BMC Plant Biol.* 17:124. 10.1186/s12870-017-1072-9 28705193 PMC5513022

[B17] Lee Y. Rubio M. C. Alassimone J. Geldner N. (2013). A mechanism for localized lignin deposition in the endodermis. *Cell* 153 402–412. 10.1016/j.cell.2013.02.045 23541512

[B18] Li B. Kamiya T. Kalmbach L. Yamagami M. Yamaguchi K. Shigenobu S. (2017). Role of LOTR1 in nutrient transport through organization of spatial distribution of root endodermal barriers. *Curr. Biol.* 27 758–765. 10.1016/j.cub.2017.01.030 28238658

[B19] Liberman L. M. Sparks E. E. Moreno-Risueno M. A. Petricka J. J. Benfey P. N. (2015). MYB36 regulates the transition from proliferation to differentiation in the *Arabidopsis* root. *Proc. Natl. Acad. Sci. U.S.A.* 112 12099–12104. 10.1073/pnas.1515576112 26371322 PMC4593085

[B20] Lim J. Helariutta Y. Specht C. D. Jung J. Sims L. Bruce W. B. (2000). Molecular analysis of the SCARECROW gene in maize reveals a common basis for radial patterning in diverse meristems. *Plant Cell* 12 1307–1318. 10.1105/tpc.12.8.1307 10948251 PMC149104

[B21] Lim J. Jung J. W. Lim C. E. Lee M. H. Kim B. J. Kim M. (2005). Conservation and diversification of SCARECROW in maize. *Plant Mol. Biol.* 59 619–630. 10.1007/s11103-005-0578-y 16244911 PMC1475827

[B22] Liska D. Martinka M. Kohanova J. Lux A. (2016). Asymmetrical development of root endodermis and exodermis in reaction to abiotic stresses. *Ann. Bot.* 10.1093/aob/mcw047 [Epub ahead of print]. 27112163 PMC5055619

[B23] Lucas M. Swarup R. Paponov I. A. Swarup K. Casimiro I. Lake D. (2011). Short-Root regulates primary, lateral, and adventitious root development in *Arabidopsis*. *Plant Physiol.* 155 384–398. 10.1104/pp.110.165126 21030506 PMC3075784

[B24] Lux A. Morita S. Abe J. Ito K. (2005). An improved method for clearing and staining free-hand sections and whole-mount samples. *Ann. Bot.* 96 989–996. 10.1093/aob/mci266 16192293 PMC4247103

[B25] Martinka M. Dolan L. Pernas M. Abe J. Lux A. (2012). Endodermal cell-cell contact is required for the spatial control of Casparian band development in *Arabidopsis thaliana*. *Ann. Bot.* 110 361–371. 10.1093/aob/mcs110 22645115 PMC3394653

[B26] Moore M. J. Soltis P. S. Bell C. D. Burleigh J. G. Soltis D. E. (2010). Phylogenetic analysis of 83 plastid genes further resolves the early diversification of eudicots. *Proc. Natl. Acad. Sci. U.S.A.* 107 4623–4628. 10.1073/pnas.0907801107 20176954 PMC2842043

[B27] Nakajima K. Sena G. Nawy T. Benfey P. N. (2001). Intercellular movement of the putative transcription factor SHR in root patterning. *Nature* 413 307–311. 10.1038/35095061 11565032

[B28] Nakayama T. Shinohara H. Tanaka M. Baba K. Ogawa-Ohnishi M. Matsubayashi Y. (2017). A peptide hormone required for Casparian strip diffusion barrier formation in *Arabidopsis* roots. *Science* 355 284–286. 10.1126/science.aai9057 28104889

[B29] Naseer S. Lee Y. Lapierre C. Franke R. Nawrath C. Geldner N. (2012). Casparian strip diffusion barrier in *Arabidopsis* is made of a lignin polymer without suberin. *Proc. Natl. Acad. Sci. U.S.A.* 109 10101–10106. 10.1073/pnas.1205726109 22665765 PMC3382560

[B30] Pant D. D. Basu N. (1997). A comparative study of the leaves of *Cathaya argyrophylla* Chun & Kuang and three species of Keteleeria Carriitre. *Bot. J. Linn. Soc.* 75 271–282. 10.1111/j.1095-8339.1977.tb01488.x

[B31] Paquette A. J. Benfey P. N. (2005). Maturation of the ground tissue of the root is regulated by gibberellin and SCARECROW and requires SHORT-ROOT. *Plant Physiol.* 138 636–640. 10.1104/pp.104.058362 15955927 PMC1150384

[B32] Peirson D. R. Dumbroff E. B. (1969). Demonstration of a complete Casparian strig in Avena and lpomoea by a fluorescent staining technique. *Can. J. Bot.* 47 1869–1871. 10.1139/b69-274

[B33] Pfister A. Barberon M. Alassimone J. Kalmbach L. Lee Y. Vermeer J. E. (2014). A receptor-like kinase mutant with absent endodermal diffusion barrier displays selective nutrient homeostasis defects. *Elife* 3:e03115. 10.7554/eLife.03115 25233277 PMC4164916

[B34] Racolta A. Bryan A. C. Tax F. E. (2014). The receptor-like kinases GSO1 and GSO2 together regulate root growth in *Arabidopsis* through control of cell division and cell fate specification. *Dev. Dyn.* 243 257–278. 10.1002/dvdy.24066 24123341

[B35] Ron M. Kajala K. Pauluzzi G. Wang D. Reynoso M. A. Zumstein K. (2014). Hairy root transformation using Agrobacterium rhizogenes as a tool for exploring cell type-specific gene expression and function using tomato as a model. *Plant Physiol.* 166 455–469. 10.1104/pp.114.239392 24868032 PMC4213079

[B36] Roppolo D. Boeckmann B. Pfister A. Boutet E. Rubio M. C. Denervaud-Tendon V. (2014). Functional and evolutionary analysis of the Casparian Strip membrane domain protein family. *Plant Physiol.* 165 1709–1722. 10.1104/pp.114.239137 24920445 PMC4119050

[B37] Roppolo D. De Rybel B. Denervaud Tendon V. Pfister A. Alassimone J. Vermeer J. E. (2011). A novel protein family mediates Casparian strip formation in the endodermis. *Nature* 473 380–383. 10.1038/nature10070 21593871

[B38] Scheres B. Benfey P. Dolan L. (2002). Root development. *Arabidopsis Book* 1:e0101. 10.1199/tab.0101 22303222 PMC3243376

[B39] Schreiber L. Franke R. B. (2011). “Endodermis and exodermis in roots,” in *eLS* (Chichester: John Wiley & Sons, Ltd). 10.1002/9780470015902.a0002086.pub2

[B40] Sena G. Jung J. W. Benfey P. N. (2004). A broad competence to respond to SHORT ROOT revealed by tissue-specific ectopic expression. *Development* 131 2817–2826. 10.1242/dev.01144 15142972

[B41] Sozzani R. Cui H. Moreno-Risueno M. A. Busch W. Van Norman J. M. Vernoux T. (2010). Spatiotemporal regulation of cell-cycle genes by SHORTROOT links patterning and growth. *Nature* 466 128–132. 10.1038/nature09143 20596025 PMC2967763

[B42] Stasovski E. Peterson C. A. (1993). Effects of drought and subsequent rehydration on the structure, vitality, and permeability of *Allium cepa* adventitious roots. *Can. J. Bot.* 71 700–707. 10.1139/b93-080

[B43] Sun H. J. Uchii S. Watanabe S. Ezura H. (2006). A highly efficient transformation protocol for Micro-Tom, a model cultivar for tomato functional genomics. *Plant Cell Physiol.* 47 426–431. 10.1093/pcp/pci251 16381658

[B44] Tomato Genome Consortium (2012). The tomato genome sequence provides insights into fleshy fruit evolution. *Nature* 485 635–641. 10.1038/nature11119 22660326 PMC3378239

[B45] Warmbrodt R. D. Evert R. F. (1979). Comparative leaf structure of several species of homosporous leptosporangiate ferns. *Am. J. Bot.* 66 412–440. 10.1002/j.1537-2197.1979.tb06242.x

[B46] Wu S. Gallagher K. L. (2014). The movement of the non-cell-autonomous transcription factor, SHORT-ROOT relies on the endomembrane system. *Plant J.* 80 396–409. 10.1111/tpj.12640 25124761

[B47] Wu S. Hayashi T. Lee C. M. Price S. Divol F. Henry S. (2014). A plausible mechanism, based upon SHORT-ROOT movement, for regulating the number of cortex cell layers in roots. *Proc. Natl. Acad. Sci. U.S.A.* 111 16184–16189. 10.1073/pnas.1407371111 25352666 PMC4234584

[B48] Wu X. Lin J. Lin Q. Wang J. Schreiber L. (2005). Casparian strips in needles are more solute permeable than endodermal transport barriers in roots of *Pinus bungeana*. *Plant Cell Physiol.* 46 1799–1808. 10.1093/pcp/pci194 16170202

[B49] Yang J. Ding C. Xu B. Chen C. Narsai R. Whelan J. (2015). A Casparian strip domain-like gene, CASPL, negatively alters growth and cold tolerance. *Sci. Rep.* 5:14299. 10.1038/srep14299 26399665 PMC4585827

[B50] Ye J. Wang X. Hu T. X. Zhang F. X. Wang B. Li C. (2017). An indel in the promoter of AL-ACTIVATED MALATE TRANSPORTER 9 selected during tomato domestication determines fruit malate contents and aluminum tolerance. *Plant Cell* 29 2249–2268. 10.1105/tpc.17.00211 28814642 PMC5635988

[B51] Yoon J. Choi H. An G. (2015). Roles of lignin biosynthesis and regulatory genes in plant development. *J. Integr. Plant Biol.* 57 902–912. 10.1111/jipb.12422 26297385 PMC5111759

[B52] Zelko I. Lux A. Sterckeman T. Martinka M. Kollarova K. Liskova D. (2012). An easy method for cutting and fluorescent staining of thin roots. *Ann. Bot.* 110 475–478. 10.1093/aob/mcs046 22419758 PMC3394640

