# Supplementary figures and images for: Spatial Expression and Functional Analysis of Casparian Strip Regulatory Genes in Endodermis Reveals the Conserved Mechanism in Tomato
Source: Front Plant Sci. 2018 Jun 22;9:832. doi: 10.3389/fpls.2018.00832 (PMC6024017; doi:10.3389/fpls.2018.00832)

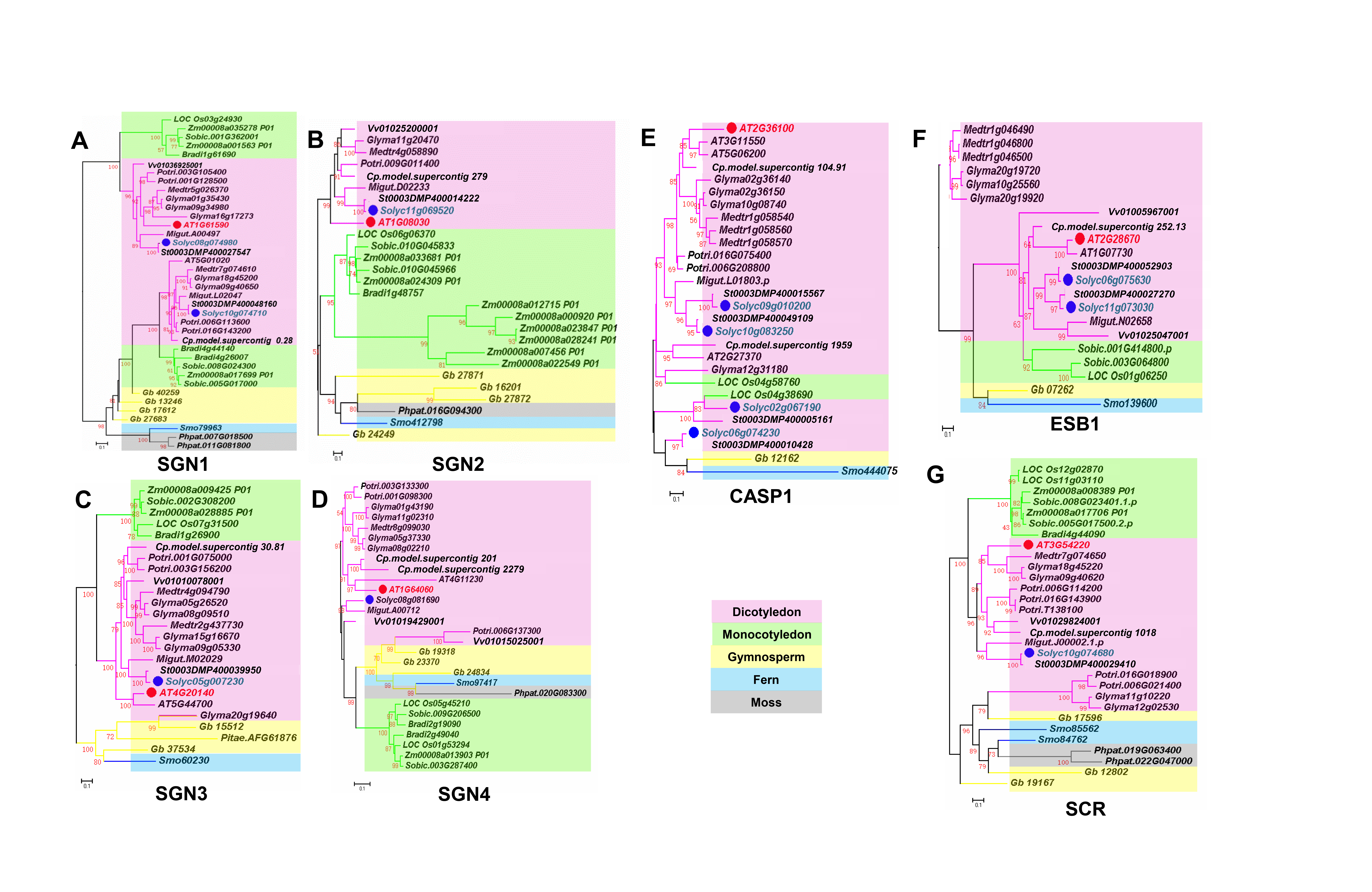

Supplement: FIGURE S1 — Phylogenetic trees of Casparian strip regulatory genes SGN1 (A), SGN2 (B), SGN3 (C), SGN4 (D), CASP1 (E), ESB1 (F) and SCR (G). See Supplementary Table S1 for the species abbreviations. [file Image_1.TIF]

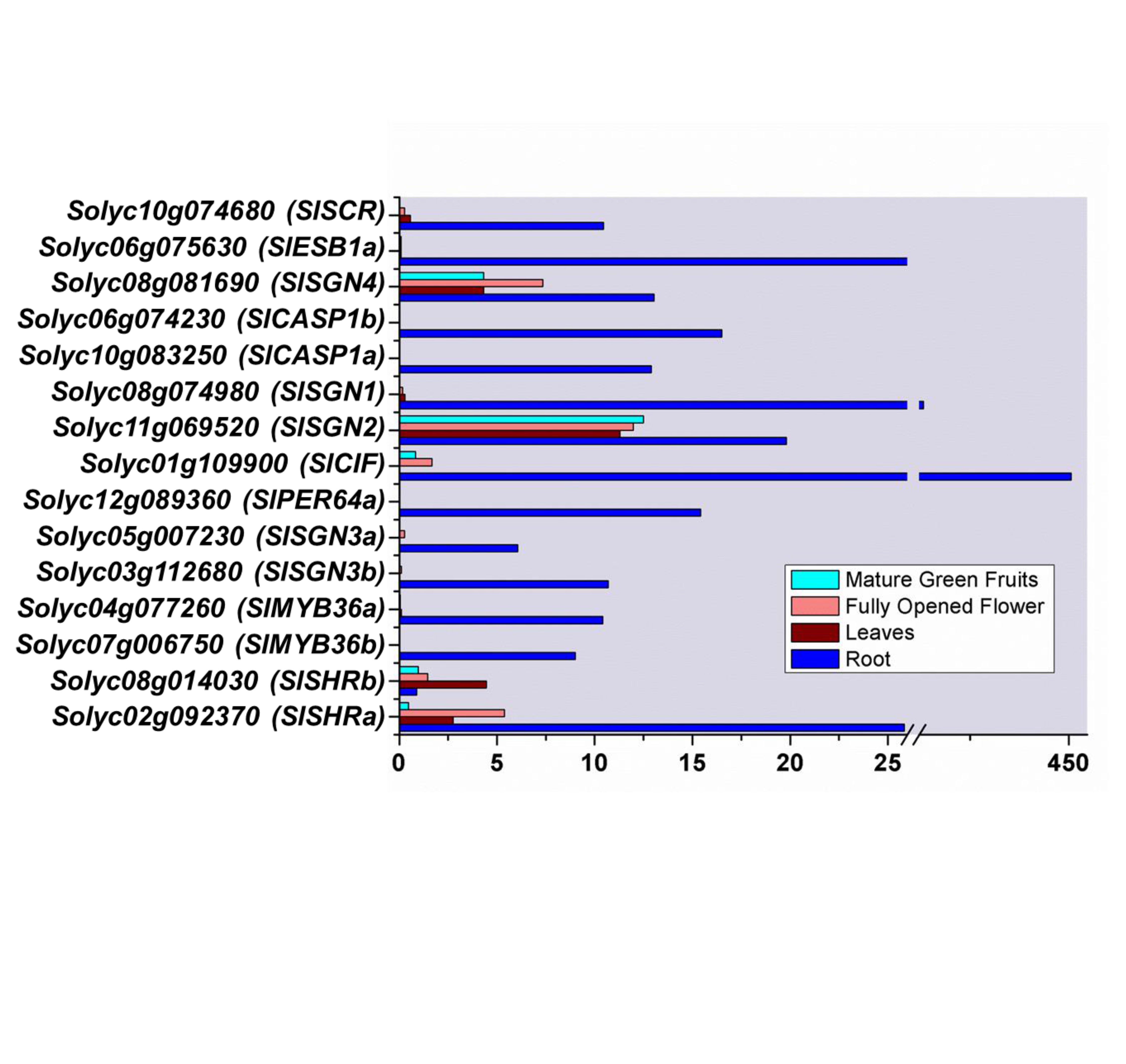

Supplement: FIGURE S2 — Normalized expression of genes in different tissues of tomato. [file Image_2.TIF]

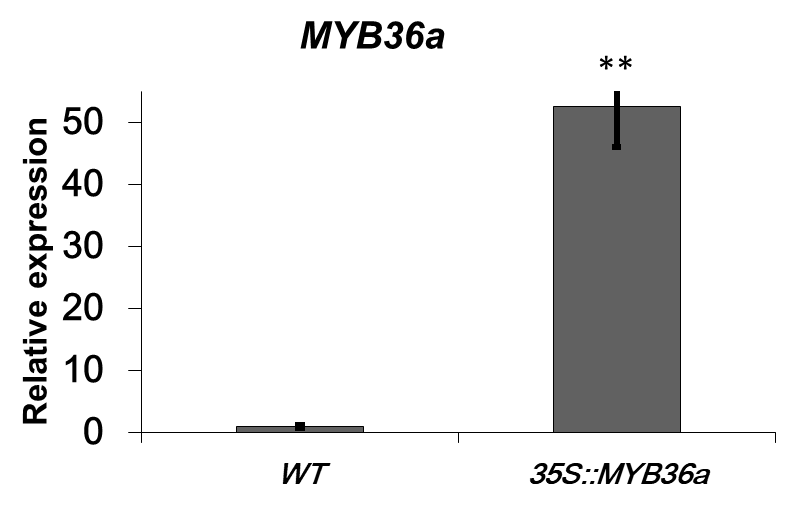

Supplement: FIGURE S3 — The MYB36a transcript levels in WT and 35S::SlMYB36a roots using qRT-PCR. Asterisks indicate significant differences from WT, ∗∗ P < 0.01, Student’s t test. Values represent the means and error bars indicate SD of the mean from three replicates. [file Image_3.TIF]

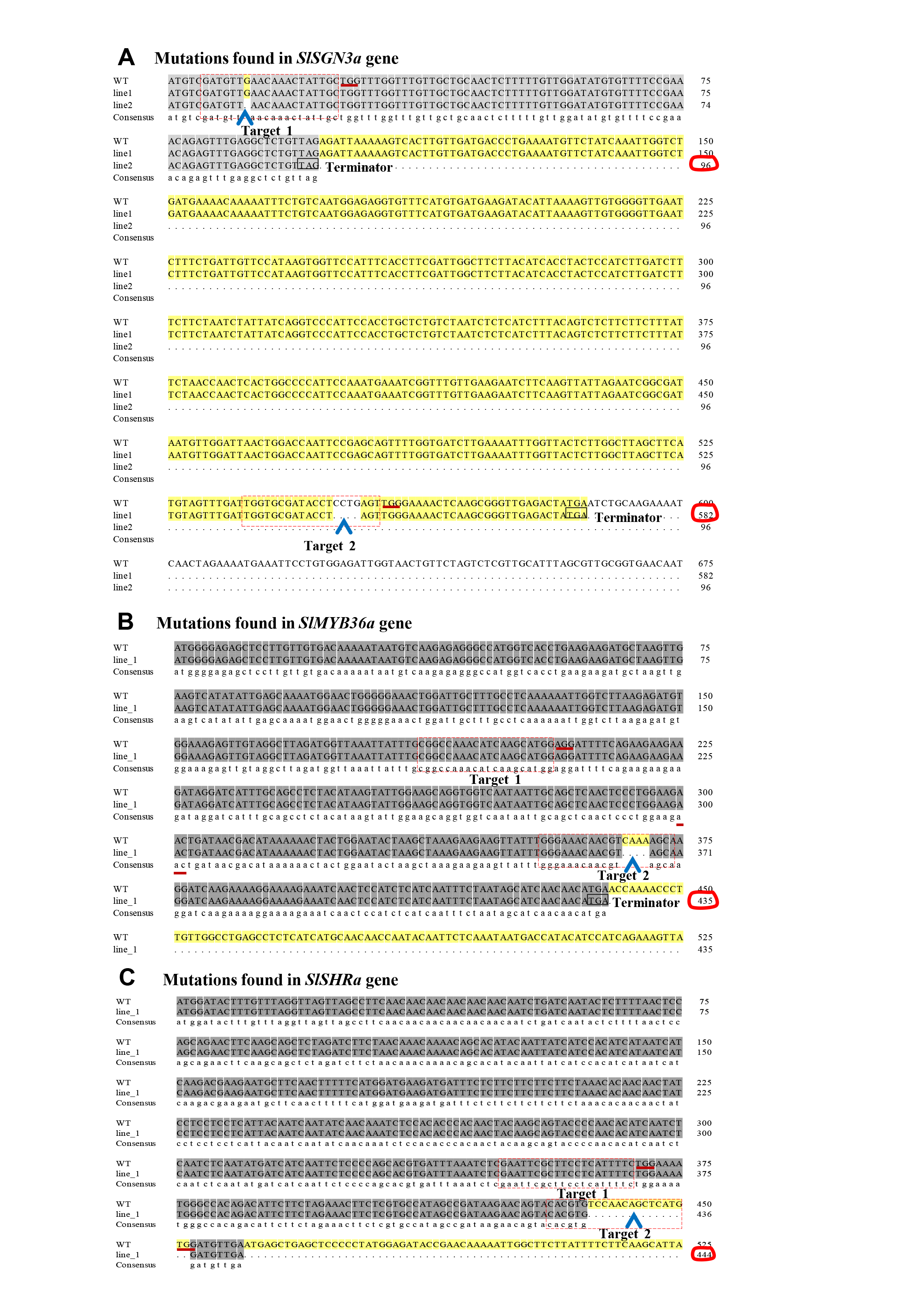

Supplement: FIGURE S4 — Alignment of sequences with Cas9-induced mutations obtained from roots transformed with CRISPR/Cas system for SlSGN3a (A), SlMYB36a (B) and SlSHRa (C). Designed target sequences are labeled in the red boxes. The sequences of mutant lines (line 1, line 2) are shown underneath the wild type (WT). The single guide RNA (sgRNA) is marked with red line and mutations are indicated by the blue arrowheads. The changes in length compared with the wild type (SlSGN3a: 3753 bp; SlMYB36a: 978bp; SlSHRa: 1524bp) are shown in red circles on the right. [file Image_4.TIF]

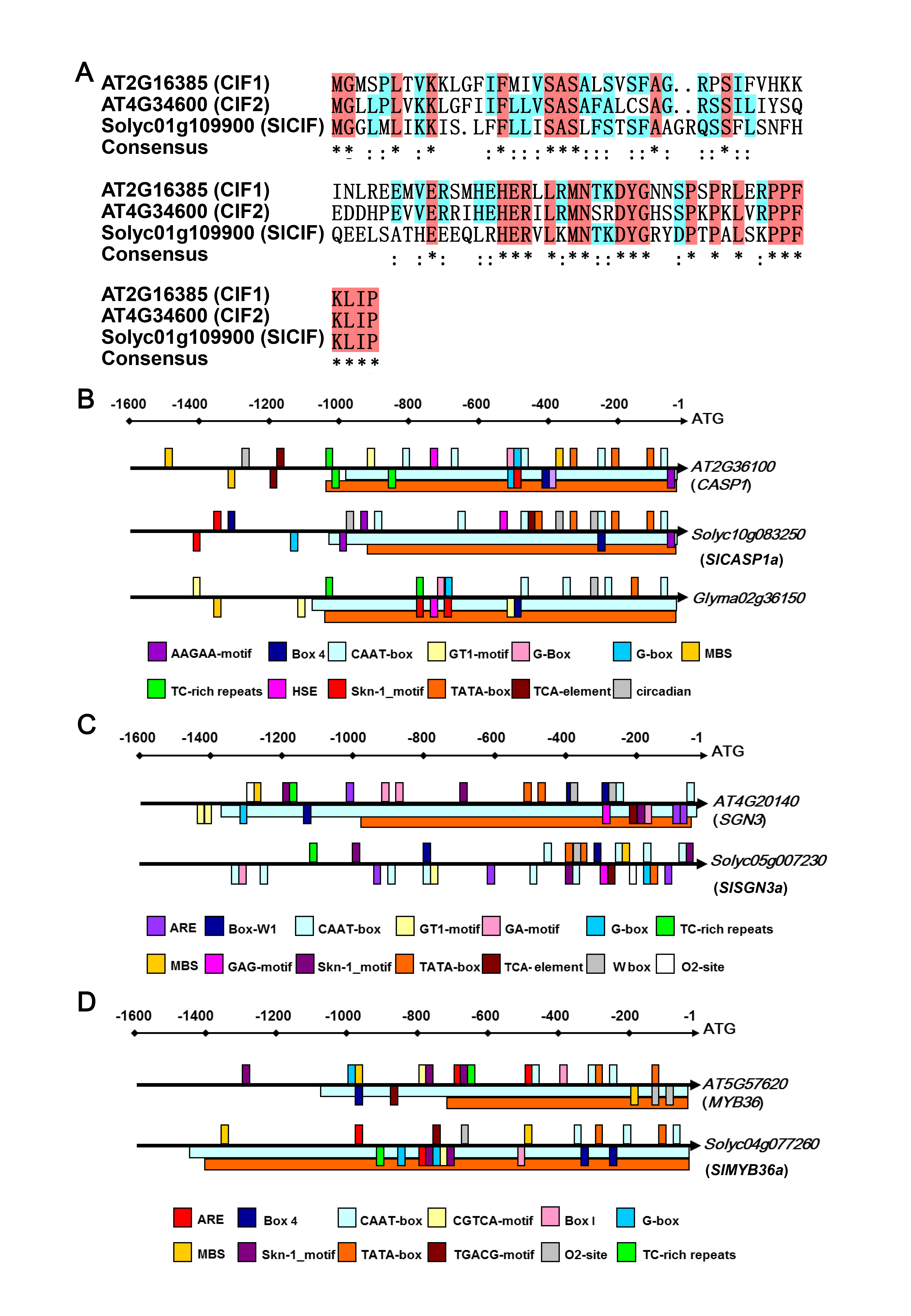

Supplement: FIGURE S5 — Amino acid sequence alignment of the peptides between Arabidopsis thaliana and tomato (A). The homology sequences are shaded in watermelon red. Conservation of promoter regions of CASP1 homologs (B), SGN3 homologs (C) and MYB36 homologs (D). Cis-acting regulatory sites present in a promoter sequence were searched using PlantCARE. [file Image_5.TIF]

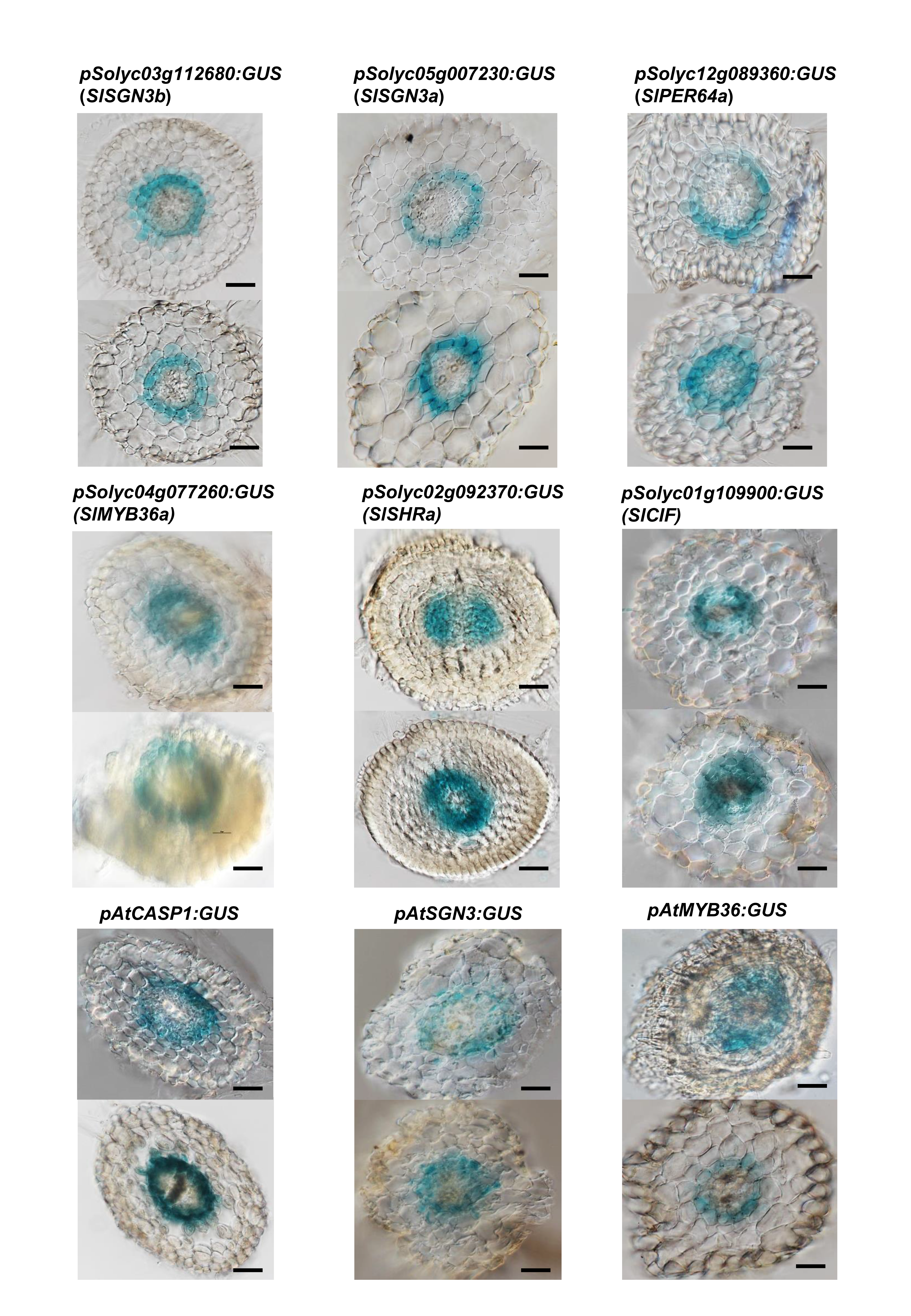

Supplement: FIGURE S6 — Cell type-specific expression in tomato conferred by the Solanum lycopersicum and Arabidopsis promoters. [file Image_6.TIF]
