# Supplementary material for: Spatial Expression and Functional Analysis of Casparian Strip Regulatory Genes in Endodermis Reveals the Conserved Mechanism in Tomato
Source: Front Plant Sci. 2018 Jun 22;9:832. doi: 10.3389/fpls.2018.00832 (PMC6024017; doi:10.3389/fpls.2018.00832)
Supplement: TABLE S1 — Numbers of homologous genes in different plant species. Species abbreviations are as follows: Potri, Populus trichocarpa; Medtr, Medicago truncatula; Glyma, Glycine max; Cp, Carica papaya; AT, Arabidopsis thaliana; Vv, Vitis vinifera; St, Solanum tuberosum; Solyc, Solanum lycopersicum; Migut, Mimulus guttatus; Bradi, Brachypodium distachyon; LOC_Os, Oryza sativa; Zm, Zea mays; Sobic, Sorghum bicolor; Gb, Ginkgo biloba; Pitae, Pinus taeda; Smo, Selaginella moellendorffii; Phpat, Physcomitrella patens. [file Table_1.PDF]

Classification	Plant Name	Abbreviations	SHR	MYB36	CIF	PER64	SGN1	SGN2	SGN3	SGN4	CASP1	ESB1	SCR
	Arabidopsis thaliana	AT	1	3	2	2	2	1	2	2	3	2	1
	Populus trichocarpa	Potri	4	4	1	1	4	1	2	3	2		5
	Medicago truncatula	Medtr	3	4	1	2	2	1	2	1	3	3	1
	Glycine max	Glyma	6	10	4	3	5	1	5	4	3	3	4
Dicotyledon	*Carica papaya*	Cp	2	3		2	1	1	1	2	2	1	1
	Vitis vinifera	Vv	2	3	1	3	1	1	1	2		2	1
	Solanum tuberosum	St	2	2	1	3	2	1	1		4	2	1
	Solanum lycopersicum	Solyc	2	2	1	4	2	1	1	1	4	2	1
	Mimulus guttatus	Migut	2	4	1	2	2	1	1	1	1	1	1
	Brachypodium distachyon	Bradi	1	2	2	1	3	1	1	2			1
Monocotyledon	*Oryza sativa*	LOC_Os	2	4	1	2	1	1	1	2	2	1	2
	Zea mays	Zm		4	1		3	8	2	1			2
	Sorghum bicolor	Sobic	2	3	1	1	3	2	1	2		2	2
Gymnosperm	*Ginkgo biloba*	Gb	1	1	1	3	4	4	2	3	1	1	3
	Pinus taeda	Pitae				1			1				
Ferns	*Selaginella moellendorffii*	Smo	2	1	1	1	1	1	1	1	1	1	2
Moss	*Physcomitrella patens*	Phpat	2				2	1		1			2
