# Supplementary material for: Spatial Expression and Functional Analysis of Casparian Strip Regulatory Genes in Endodermis Reveals the Conserved Mechanism in Tomato
Source: Front Plant Sci. 2018 Jun 22;9:832. doi: 10.3389/fpls.2018.00832 (PMC6024017; doi:10.3389/fpls.2018.00832)
Supplement: TABLE S2 — Primers used for molecular cloning. [file Table_2.PDF]

Primer name	Sequence	Note
SlPSHRa-hindIII-F	CGACGGCCAGTGCCAAGCTTGGGGAGGTGTAAAACAGATTG	Promoter
SlPSHRa-psiI-R	ACAAAGTTGGCATTATAATTAACACTAAAAGGGTTACGTATCT	Promoter
SlPMYB36a-hindIII-F	CGACGGCCAGTGCCAAGCTTTGTATATCTTCGTGTTATACACT	Promoter
SlPMYB36a-psiI-R	ACAAAGTTGGCATTATAATTCTTGATATTTTCACTAACAATATTG	Promoter
SlPER64a-hindIII-F	CGACGGCCAGTGCCAAGCTTTCGATGACCTTATTAAAACGAC	Promoter
SlPER64a-psiI-R	ACAAAGTTGGCATTATAAAACTAATTCTGTTTGAAAAATTAGT	Promoter
SlPSGN2-hindIII-F	CGACGGCCAGTGCCAAGCTTTGGAAATAAATGGGCTTTTACGT	Promoter
SlPSGN2-psiI-R	ACAAAGTTGGCATTATAACAATCTATCCCAGAAATCAAACTG	Promoter
SlPSGN3b-hindIII-F	CGACGGCCAGTGCCAAGCTTAATTGGAGGTAATAATGGATGTG	Promoter
SlPSGN3b-psiI-R	ACAAAGTTGGCATTATAAGTTAATATTTGTGAATGGAACACAA	Promoter
SlPSGN3a-hindIII-F	CGACGGCCAGTGCCAAGCTTGTTTGGAGTCAACTATCACTATGG	Promoter
SlPSGN3a-psiI-R	ACAAAGTTGGCATTATAAGATTTGAAAGTCACTATGAAAAACT	Promoter
SlPCIF-hindIII-F	CGACGGCCAGTGCCAAGCTTGGATTGCCCAAGTCTTACAAG	Promoter
SlPCIF-psiI-R	ACAAAGTTGGCATTATAACTCTGGCACTCTTATTATTTTTAGG	Promoter
pAtCASP1-hindIII-F	CGACGGCCAGTGCCAAGCTTTTAATCTGCATAAAAGTGAGTATGAG	Promoter
pAtCASP1-psiI-R	ACAAAGTTGGCATTATAATGTGTTGTGTATTGGAATGTTTTGT	Promoter
pAtSGN3-hindIII-F	CGACGGCCAGTGCCAAGCTTCTGAGTGAGATTCATACTTGGTGC	Promoter
pAtSGN3-psiI-R	ACAAAGTTGGCATTATAAGTTTTCTTCTTCGTCGTCTTATG	Promoter
pAtMYB36-hindIII-F	CGACGGCCAGTGCCAAGCTTCCCACCTCTCAAAACAATAAAT	Promoter
pAtMYB36-psiI-R	ACAAAGTTGGCATTATAACTTGTCGTTGTTGTTCTCTTCC	Promoter
SlSHRa-attb1-F	AAAAAGCAGGCTTCATGGATACTTTGTTTAGGTTAGTTAGC	Gene
SlSHRa-attb2-R	AGAAAGCTGGGTTAGGCTTCCATGCACTGG	Gene
SlMYB36a-attb1-F	AAAAAGCAGGCTTCATGGGGAGAGCTCCTTG	Gene
SlMYB36a-attb2-R	AGAAAGCTGGGTTTTGTTCCCTGGGGTATCTTAG	Gene
Cri-SlSHRa-F	GAATCTAACAGTGTAGTTTGGAATTCGCTTCCTCATTTTCGTTTTAGAGCT AGAAATAG	CRISPR
Cri-SlSHRa-R	GCTATTTCTAGCTCTAAAACCATGAGCTGTTGGACACGTGCAAACTACAC TGTTAGATT	CRISPR
Cri-SlMYB36a-F	GAATCTAACAGTGTAGTTTGCGGCCAAACATCAAGCATGGGTTTTAGAG CTAGAAATAG	CRISPR
Cri-SlMYB36a-R	GCTATTTCTAGCTCTAAAACTGCTTTTGACGTTGTTTCCCCAAACTACACT GTTAGATT	CRISPR
Cri-SlSGN3a-F	GAATCTAACAGTGTAGTTTGGATGTTGAACAAACTATTGCGTTTTAGAGC TAGAAATAG	CRISPR
Cri-SlSGN3a-R	GCTATTTCTAGCTCTAAAACACTCAGGAGGTATCGCACCACAAACTACAC TGTTAGATT	CRISPR
