# Supplementary material for: Spatial Expression and Functional Analysis of Casparian Strip Regulatory Genes in Endodermis Reveals the Conserved Mechanism in Tomato
Source: Front Plant Sci. 2018 Jun 22;9:832. doi: 10.3389/fpls.2018.00832 (PMC6024017; doi:10.3389/fpls.2018.00832)
Supplement: TABLE S3 — Cell layers in WT, 35S::SlSHRa, and slshra. [file Table_3.PDF]

WT	*35S::SlSHRa*	*slshra*
4	8	2
4	9	3
3	10	3
4	6	2
4	11	2
4	8	3
5	7	3
4	10	3
5	9	3
4	8	3
3	8	3
5	8	2
4	8	3
4	7	2
5	9	2
4	10	3
4	7	3
4	8	3
3	9	3
4	7	3
